# BrcaDx: precise identification of breast cancer from expression data using a minimal set of features

**DOI:** 10.3389/fbinf.2023.1103493

**Published:** 2023-05-23

**Authors:** Sangeetha Muthamilselvan, Ashok Palaniappan

**Affiliations:** Department of Bioinformatics, School of Chemical and Biotechnology, SASTRA University, Thanjavur, Tamil Nadu, India

**Keywords:** breast cancer, predictive diagnosis, principal component analysis, k-means clustering, transcriptomics, biomarker discovery, progression-significant genes, stage-informed models

## Abstract

**Background:** Breast cancer is the foremost cancer in worldwide incidence, surpassing lung cancer notwithstanding the gender bias. One in four cancer cases among women are attributable to cancers of the breast, which are also the leading cause of death in women. Reliable options for early detection of breast cancer are needed.

**Methods:** Using public-domain datasets, we screened transcriptomic profiles of breast cancer samples, and identified progression-significant linear and ordinal model genes using stage-informed models. We then applied a sequence of machine learning techniques, namely, feature selection, principal components analysis, and k-means clustering, to train a learner to discriminate “cancer” from “normal” based on expression levels of identified biomarkers.

**Results:** Our computational pipeline yielded an optimal set of nine biomarker features for training the learner, namely, NEK2, PKMYT1, MMP11, CPA1, COL10A1, HSD17B13, CA4, MYOC, and LYVE1. Validation of the learned model on an independent test dataset yielded a performance of 99.5% accuracy. Blind validation on an out-of-domain external dataset yielded a balanced accuracy of 95.5%, demonstrating that the model has effectively reduced the dimensionality of the problem, and learnt the solution. The model was rebuilt using the full dataset, and then deployed as a web app for non-profit purposes at: https://apalania.shinyapps.io/brcadx/. To our knowledge, this is the best-performing freely available tool for the high-confidence diagnosis of breast cancer, and represents a promising aid to medical diagnosis.

## Introduction

Breast cancer is the most commonly diagnosed cancer in the world, with a staggering 2.3 million cases in 2020 (Sung et al., 2021). It accounts for approximately 24.5% of cancer cases and 15.5% of cancer deaths among women, ranking #1 in both incidence and mortality in most countries. Modelling studies predict an exponential and asymmetric rate of increase in breast cancer incidence among low human development index (HDI) nations relative to high HDI nations, due to an unmitigated increase in risk factors in low HDI nations (Soerjomataram and Bray, 2021). In India, for, e.g., the age of onset of breast cancer has advanced 10 years earlier relative to that in Europe and America. About 29%–52% of women with breast cancer in India present in the more severe advanced stages, leading to poor prognosis (Bhattacharyya et al., 2020). Low HDI nations are likely to also suffer from problems due to the lack of social awareness and existent taboos, especially in rural areas. Alternative diagnostic methods based on a minimal set of biomarkers are urgently needed to effectively redress the situation (Duan et al., 2016).

The advent of–omics data has ushered in AI-based approaches to cancer diagnosis. However, contemporary AI-based diagnostic methods are saddled with unreasonable dimensionality of the hypothesis space, and typically require sequencing of hundreds of biomarkers to achieve clinical utility. Dimensionality reduction techniques like principal components (PC) analysis are generally used for extracting optimal feature subsets, especially when linear relationships exist in the dataset. PC analysis has been earlier used to detect multiple cancer types simultaneously, with a costly compromise in accuracy and interpretation (Fakoor et al., 2013). Working in the space of PCs tends to lead to more robust clustering outcomes (Ding and He, 2004), and k-means clustering is an effective technique for analyzing transformed spaces (Berkhin et al., 2006; Raykov et al., 2016). Building on the above observations, this study has two principal objectives: 1) develop and validate the most efficient integrative computational pipeline for breast cancer classification based on a minimal hypothesis space; and 2) translate the resulting diagnostic classifier into a web-app service to aid medical decision-making.

## Materials and methods

**FIGURE 1:**
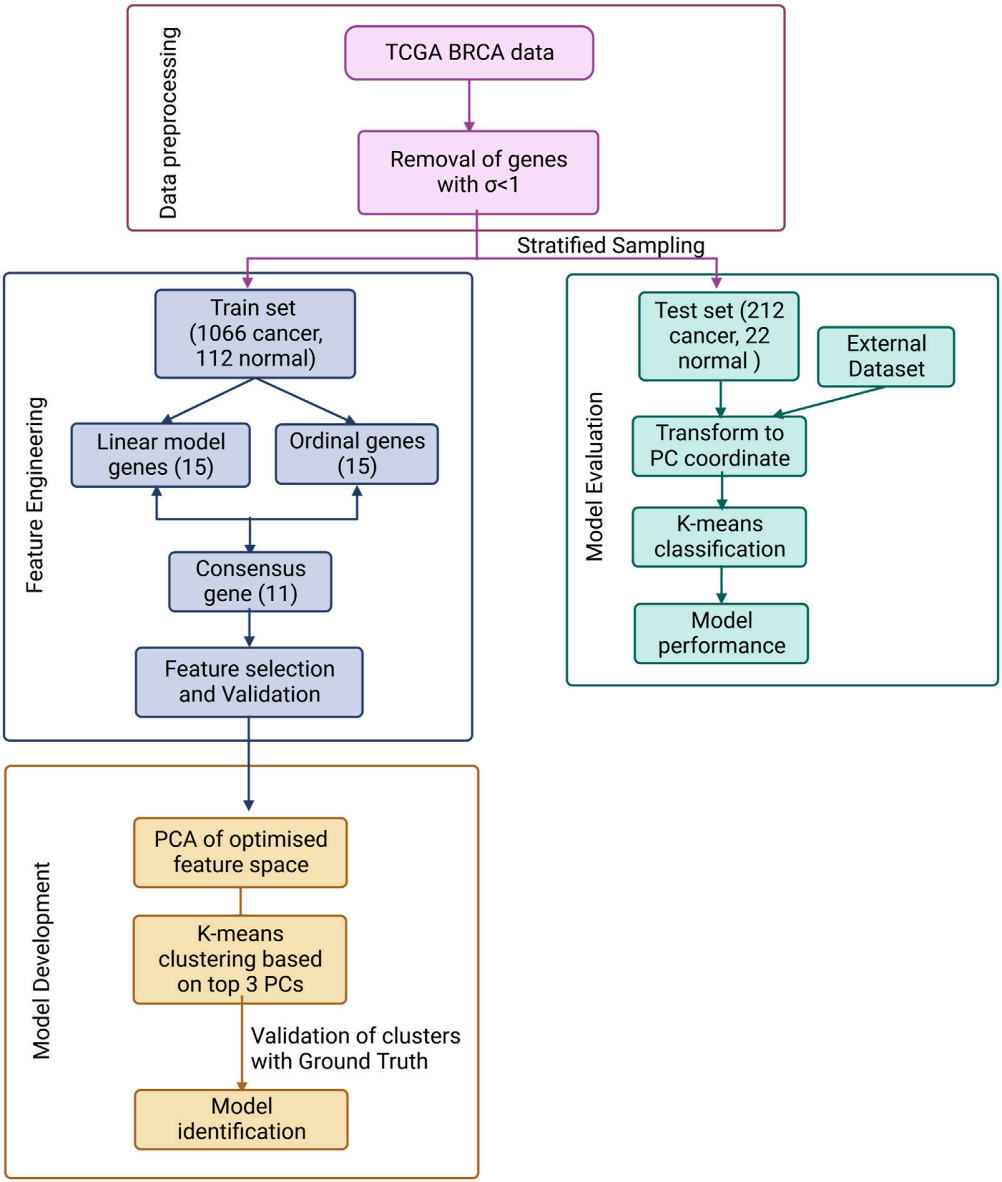
ML pipeline used in the study for the design of a simple, effective and optimal cancer vs. normal classifier.

The overall workflow is summarised in Figure 1 and discussed in detail below.

### Data pre-processing

RSEM-normalised BRCA expression dataset (gdac.broadinstitute.org_BRCA.Merge_rnaseqv2__illuminahiseq_rnaseqv2__unc_edu__Level_3__RSEM_genes_normalized__data.Level_3.2016012800.0.0. tar.gz) was retrieved from the TCGA using firebrowse portal (Deng et al., 2017) by selecting the Cohort as “Breast invasive carcinoma.” The samples were annotated as “normal” or “cancer” based on the sample-encoding part in the patient barcode (uuid) in the variable “Hybridization REF.” The sample stage was extracted from the attribute “patient.stage_event.pathologic_stage” in the associated clinical metadata file retrieved for the same cohort as gdac. broadinstitute.org_BRCA.Merge_Clinical.Level_1.2016012800.0.0. tar.gz. Genes with minimal variation in expression across the samples were removed if the expression σ < 1. The resulting data matrix was then processed through voom in limma to prepare for linear modelling (Ritchie et al., 2015). Then it was split into train: test datasets in the ratio 80:20 stratified on the target class. Data pre-processing was done in R (www.r-project.org).

### Feature engineering

The training dataset was used to identify the features for the problem. Two models were considered to extract potential features:

1) A linear model of stagewise expression in each gene was performed using R *limma* (Ritchie et al., 2015), with the following equation

$$Y=\alpha +\beta_{1}x_{1}+\beta_{2}x_{2}+\beta_{3}x_{3}+\beta_{4}x_{4}$$ (1)

where the intercept α is the baseline expression obtained from the controls, the independent variables are indicator variables of the sample’s stage, and β_i_ are the predicted log fold-change (lfc) coefficients relative to controls. Further the model was subjected to empirical Bayes adjustment for obtaining moderated t-statistics (McCarthy and Smyth, 2009). Multiple hypothesis testing was corrected using the Benjamini Hochberg method (Haynes et al., 2013).

2) An ordinal model of gene expression was also considered. Here the cancer stage is treated as a numeric variable according to the equation:

Y=aX+b (2)

where X is the cancer stage taking the values 0, 1, 2, 3, and 4, corresponding to Control, Stage-1, Stage-2, Stage-3, and Stage-4, respectively.

### Feature space optimization

Genes from the linear and ordinal expression models were ranked based on the adj. *p*-value. The consensus set between the top-ranked 15 genes of the linear and ordinal models was determined and then subjected to feature selection using Boruta (Kursa and Rudnicki, 2010) and Recursive Feature Elimination (Kuhn, 2008) (RFE). Boruta implements a wrapper algorithm based on Random Forest to select features either strongly or weakly connected to the outcome variable, while RFE implements a backward selection process to identify an optimal set of predictors. Post feature-selection, the retained features were validated using variance inflation analysis, involving regressing each independent variable on all the other independent variables in turn, identifying and removing redundancy till a minimal feature space has been obtained (Ferré et al., 2009). The variance inflation factor (VIF) score was calculated using:

$$VIF=\frac{1}{1-R^{2}}$$ (3)

where *R* ^2^ is the goodness-of-fit of the fitted model. A variable with VIF = 1.0 is perfectly independent of all other variables, whereas any variable with VIF >2.0 was deemed multicollinear with the other variables and iteratively eliminated.

### PCA-based K-Means clustering

From the validated set of features, the principal components of the subspace spanned by these features were found, and the optimal number of principal components identified using three different criteria, namely, scree plot, Kaiser-Guttmann rule (Kaiser, 1992), and the proportion of variance explained. K-means clustering with k = 2 was performed in the space defined by the optimal principal components, to examine separation between the normal and cancer samples.

### Model evaluation

Classification performance from clustering in the principal components space was evaluated using metrics like accuracy, precision, recall, F_1_-score, area under Receiver Operating Characteristic curve (AUROC) and the Matthews correlation coefficient (MCC) (Chicco and Jurman, 2020). Balanced accuracy is a class-weighted measure of accuracy, reporting the average performance on both the diagnostic classes. F1-score is defined as the harmonic mean of the precision and recall. Performance evaluation was done using the test dataset, and an independent external dataset, namely, “BRCA-KR” retrieved from the ICGC DataPortal (https://dcc.icgc.org/). BRCA-KR had just three control samples, hence it was augmented with 218 control samples from GTEx for the purposes of evaluation (GTEx, 2017).

## Results

BRCA RNA-Seq data retrieved from TCGA consisted of 1,212 samples each with the expression values of 20,532 genes. Post data pre-processing, we obtained a dataset of 1,178 samples, 18,880 genes. We performed an 80:20 stratified sampling of the dataset (with 1,066 cancer, 112 normal samples) based on the outcome class to obtain the training dataset (with 854 cancer, 90 normal samples), and test dataset (with 212 cancer, 22 normal samples). The training dataset was voom-processed using limma and then subjected to the two modeling protocols. At an adj. *p*-value threshold of 1E-5, the linear model yielded 8,961 significant genes (Supplementary File S1), while the ordinal model yielded 6,888 significant (Supplementary File S2). We examined the overlap among the top 15 genes from each model, which produced eleven consensus genes for subsequent analysis.

**FIGURE 2:**
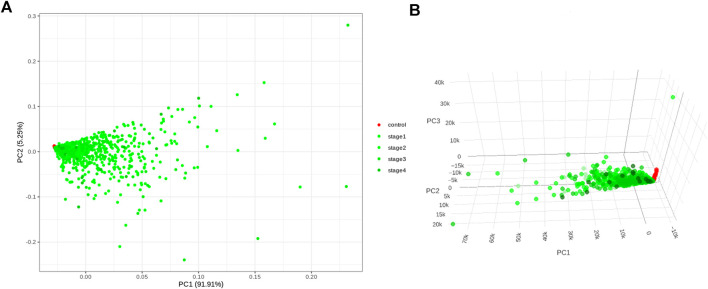
PC analysis of the biomarker expression space. With **(A)** top two components; and **(B)** top three components. It is seen that the use of three components expands the separation between the cancer samples and controls in better-defined sub-spaces.

**FIGURE 3:**
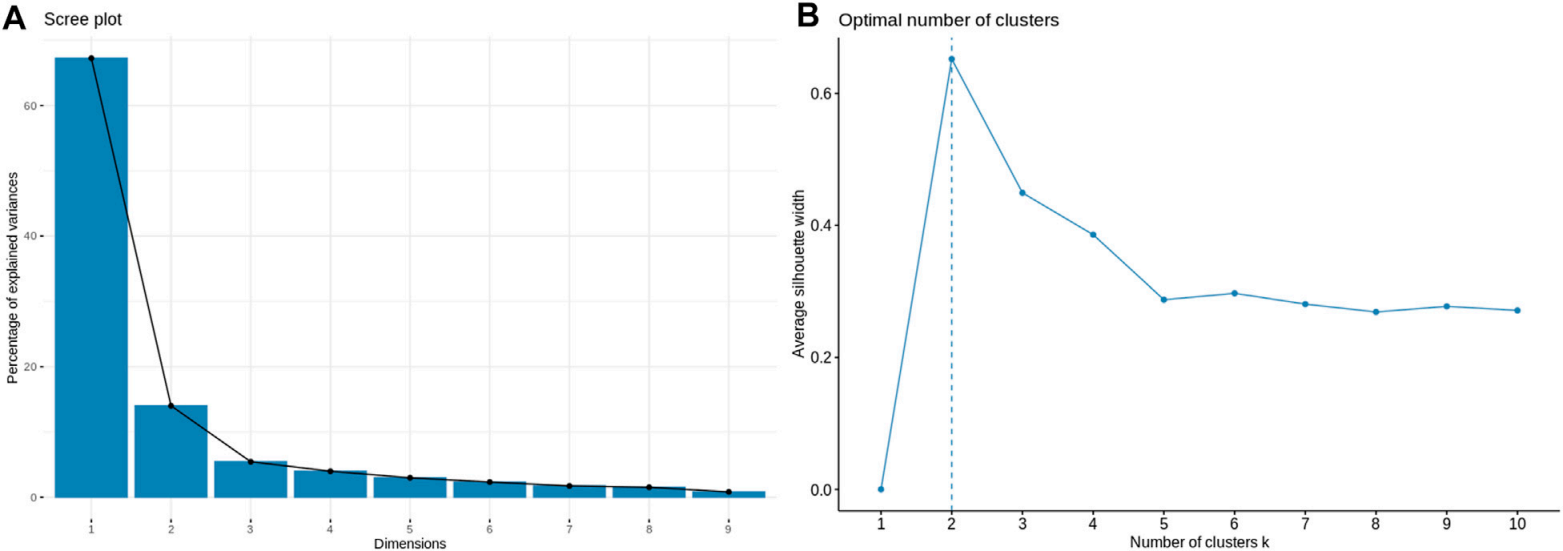
Model parameterization. **(A)** Scree plot for determination of the optimal number of principal components. The elbow method yields the first three PCs which have a cumulative variance >85%. **(B)** Silhouette plot for ascertaining the optimal number of clusters in the structure of the transformed PC-space. The emergent value, *k* = 2, is in sync with the type of problem at hand: binary classification.

Application of the Boruta feature selection protocol on the eleven genes yielded a hypothesis space of only nine genes, while application of the RFE feature selection protocol didn’t yield any reduction in the size of the hypothesis space. A summary of the final nine consensus genes is presented in Table 1. The hypothesis space was subjected to VIF analysis, to ensure absence of multicollinearity among features, and establish a minimal non-redundant set of features (Table 1, last column). We identified the nine principal components (PCs) of this 9-dimensional space (Table 2), and then visualized the training samples using the top PCs from this analysis (Figure 2). The application of three PCs clearly resolves and separates the cancer and normal samples (Figure 2B). To decisively identify the optimal number of PCs, we examined the three criteria outlined in Methods: 1) Kaiser-Guttman criterion yielded top six PCs; 2) Scree plot showed the first three principal components to be optimal (Figure 3A); and 3) the first three PCs explained >85% variance, passing the proportion of variance explained condition. We reconciled the above findings, and chose the first three principal components to define a 3-dimensional space for applying k-means clustering. Next, we optimized the number of clusters (k) for k-means clustering using the silhouette method (RousseeuwSilhouettes, 1987) (Figure 3B). A value of k = 2 was obtained, which synchronized with the larger objective to partition the structure of the space into cancer and normal signatures.

**TABLE 1:** Summary of the consensus features from the two modeling protocols. All features are exceedingly differentially expressed with extreme significance. The largest VIF score does not exceed 1.57, corresponding to a multivariate “correlation coefficient” < 0.6.

S.No	Feature	lfc	Adj.*p*.value—linear	Adj.P.value—ordinal	Regulation status	VIF score
1	NEK2	4.57	2.94E-146	6.25E-61	UP	1.05
2	PKMYT1	4.47	1.53E-127	6.14E-53	UP	1.05
3	MMP11	5.99	3.26E-134	2.02E-53	UP	1.00
4	CPA1	−4.20	1.61E-138	2.62E-49	DOWN	1.54
5	COL10A1	7.12	2.04E-137	5.62E-54	UP	1.00
6	HSD17B13	−4.86	5.67E-117	3.71E-51	DOWN	1.22
7	CA4	−6.93	8.41E-127	9.92E-50	DOWN	1.57
8	MYOC	−6.53	3.30E-133	4.03E-57	DOWN	1.34
9	LYVE1	−4.91	3.10E-128	3.21E-47	DOWN	1.02

**TABLE 2:** Summary of the nine components from the PC analysis, ranked by associated eigenvalue. Cumulative variance enables the application of the “proportion of variance explained” criterion.

S.No	PC	Eigenvalue	Variance explained (%)	Cumulative variance explained (%)
1	PC1	34.487	67.24	67.24
2	PC2	7.181	14.00	81.24
3	PC3	2.787	5.43	86.67
4	PC4	2.039	3.97	90.65
5	PC5	1.521	2.97	93.62
6	PC6	1.191	2.32	95.94
7	PC7	0.887	1.73	97.67
8	PC8	0.781	1.52	99.19
9	PC9	0.415	0.81	100

**FIGURE 4:**
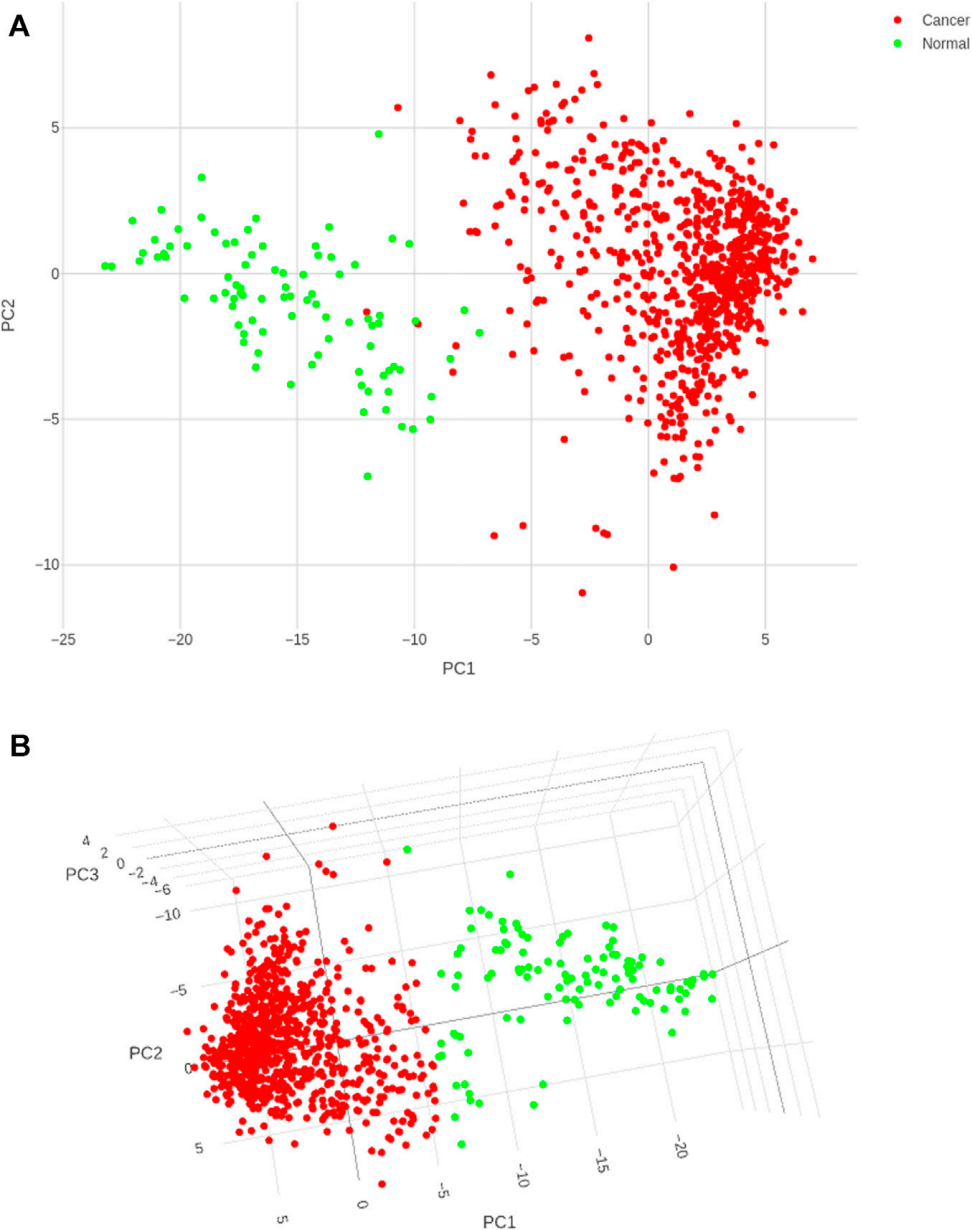
Cancer (red) and control (green) clusters obtained after training the k-means classifier. **(A)** Two-dimensional projection onto the first two principal components shows some uncertainty in the boundaries of the two clusters; **(B)** Visualization in the three-dimensional space of the PCs satisfactorily resolves the cluster boundaries.

The classifier was built using the training dataset, with 5-fold cross-validation. From Figure 4, it is clear that the k-means classifier in the 3-dimensional PC space of the identified biomarkers determinately partitioned the diagnostic space into cancer vs. normal. The prediction of the clustering outcomes was assessed against the ground truth labels in the training, test and external datasets, and presented in Table 3. It is seen that the model produced by the workflow yielded balanced accuracies of 99.53% and 95.52% on the internal validation and external validation datasets respectively. A superior MCC value was obtained for the external validation dataset, indicating the classifier has avoided any overfitting and successfully generalized the solution to the problem.

**TABLE 3:** Performance metrics of the developed k-means model in the transformed PC space of the identified nine biomarker features. Bal. acc. refers to balanced accuracy. Sensitivity is identical to the recall values. Values for the training dataset refer to 5-fold cross-validation outcomes.

S.No	Dataset	Bal. acc	Specificity	Precision	Recall	F_1_-score	AUROC	MCC
1	Training	98.83	100	100	97.66	98.81	0.909	0.89
2	Test	99.53	100	100	99.06	99.53	0.995	0.91
3	External	95.52	99.55	97.73	91.49	94.51	0.955	0.93

### Deployment

To convert the outcomes in effectively classifying cancer vs. normal based on the expression of just a handful of features, we have developed an app, BrcaDx, to freely provide the service to the academic community, based on R Shiny (Chang et al., 2023). BrcaDx is deployed at: https://apalania.shinyapps.io/brcadx/. The model was rebuilt using the full dataset for maximum discriminative performance. Based on an input of the expression values of the nine biomarkers, the app carries out the necessary log_2_ preprocessing of the values, and transforms them into the three-dimensional PC space. The transformed coordinates are fed to the learned k-means clustering model, which locates the sample in either of the two clusters, thus predicting the class of the sample. The app accepts a single-sample input as well as batch inputs (samples x biomarkers), where it accepts multiple samples, and predicts the diagnostic class for all the samples. To facilitate strictly not-for-profit applications, a video tutorial for using the app has been provided on the landing page.

## Discussion

It is significant to note that some of the biomarkers identified in our study are part of marketed and commercially available signature panels used in the context of breast cancer diagnosis and treatment. Specifically: 1) NEK2 is a constituent of the 11-gene Breast Cancer Index signature used to estimate recurrence (Zhang et al., 2013); and 2) MMP11 is a constituent biomarker of the 50-gene Prosigna (Parker et al., 2009), and 21-gene OncotypeDX (Cronin et al., 2007) signature panels, which are both used in estimating likelihood of recurrence. It is interesting to note that the Prosigna panel is based on the PAM50 signature, which is also used to subtype breast cancer into Luminal-A, Luminal-B, HER2-enriched and Basal-like (Bastien et al., 2012).

The consensus genes used to build our model are known to play key roles in cancers of the breast and other tissues, contributing to breast-cancer specific pathways as well as cancer hallmark processes (Hanahan and Weinberg, 2011). The genes NEK2, PKMYT1, and CA4 are known to play indispensable roles in cell cycle progression (Mueller et al., 1995; Lagadic-Gossmann et al., 2004; Fang and Zhang, 2016). NEK2 is documented to be overexpressed in breast-cancer tissue relative to normal tissue (Hayward et al., 2004; Cappello et al., 2014), and is required for the growth, maintenance and survival of the transformed cell (Lee and Gollahon, 2013). PKMYT1 overexpression is known to be significantly correlated with BRCA subtypes, and indicative of poor prognosis (Liu et al., 2020). Downregulation of CA4 is associated with poor prognosis in cancers other than that of the breast, notably uveal melanoma, renal cell cancer, glioma, and lung adenocarcinoma (Liu et al., 2020; Xu et al., 2020), hinting its role in hallmark processes common to many cancers, and its potential significance in establishing such hallmarks in breast cancer progression. Hypermethylation of the CPA1 gene in breast cancer cells has been earlier demonstrated (Chen et al., 2017; DeVaux and Herschkowitz, 2018), which could lead to its significant downregulation noted here. Recently, COL10A1 was identified as an overexpressed predictive biomarker for breast cancer coexpressed with LRRC15 (Fleischer et al., 2014). COL10A1 protein is a known extracellular matrix molecule released into the blood, and increased levels of circulating COL10A1 protein has been suggested as a diagnostic marker of breast cancer (Zhang et al., 2020). MYOC has been previously reported as a topranked downregulated gene in breast cancer (Giussani et al., 2018). MMP11 overexpression in early stages is necessary for cancer progression via inhibition of apoptosis, and promotion of invasion and metastasis (Li et al., 2018). Overexpression of LYVE1 has been suggested as a reliable marker of lymphatic metastasis in breast cancer patients (Zhang et al., 2015). HSD17B13 is involved in estrogen biosynthesis (Doan et al., 2014), and its tumor suppressor role in hepatocellular carcinoma has been documented (Wang et al., 2019), suggesting analogous key roles specific to breast cancer progression.

Due to the substantial heterogeneity in breast cancer, large feature spaces have been necessary for acceptable performance in contemporary classification strategies. Some of these have mandated whole genome sequencing to completely cover the biomarker space of interest (Elbashir et al., 2019). For, e.g, Zhao et al. (2020) identified 817 features and used them to build a model that achieved accuracies of 86.96% and 72.46% in different external validation datasets respectively. Mostavi et al. (2020) used a feature space of 2090 genes for discriminating cancer vs. normal, of which 323 biomarkers were designated for the task of subtyping breast cancer. Convolution-based deep neural networks (CNNs) have been applied to learn from image datasets of mammography, computed tomography (CT), magnetic resonance (MR) and histopathological slides (Saha et al., 2018; Munir et al., 2019; Jiang et al., 2020). CNNs have been used to extract features from whole-slide tissue-biopsy images, which were subsequently used to train a Support Vector Machine classifier of cancer vs. normal, yielding an accuracy of 83.3% (Araújo et al., 2017). CNNs have also been used to build models from breast ultrasound images, yielding an internal test-set performance of 92.5% accuracy, but external validation was not reported (Muduli et al., 2022). Radiogenomics approaches based on multimodal datasets have also been developed for breast cancer diagnosis (Du et al., 2022). The use of large feature spaces hinders the interpretation of these models, induces overfitting, and discourages the adoption of AI-assisted diagnosis in medical decision-making. One approach in this direction has been to use machine learning models with different feature selection algorithms such as SVM-RFE with Particle swarm optimisation (PSO), SVM-RFE with Grid search (GS), SVM-RFE with Genetic algorithm (GA), Random forest feature selection (RFFS), Random forest feature selection and grid search (RFFS-GS), and minimal redundancy maximal relevance (MRMR), of which SVM-RFE-PSO performed best with six features and 91.68% accuracy (Zhang et al., 2018). Very recently Taghizadeh et al. (2022) have advanced a solution to the “cancer” vs. “normal” problem, proposing a panel of 20 biomarkers for discriminating breast cancer from normal sample. Their study has been validated on an internal test set with a balanced accuracy ∼86%, but no external validation has been provided. Furthermore their models have not been made available for wider use. It is notable that there is zero overlap between the biomarkers identified in their study and those identified herein, indicating the orthogonal approaches used. Our study provides a reliable, interpretable, and validated generalization to the present situation, with a balanced-accuracy performance >95% on the external validation, and open-access web-server for diagnostic decision support.

## Conclusion

In this work, we set out to negotiate the compromise between model complexity and performance, and develop the simplest possible best-performing model of breast cancer classification. The designed computational pipeline yielded a novel non-redundant hypothesis space of nine biomarkers, which was transformed into a space defined by an optimal number of principal components. A k-means clustering model trained in this transformed space was able to discriminate cancer from normal samples with a high balanced accuracy of 99.5% and 95.5% on the internal and external validation datasets, respectively. At the same time, we note that the model had limited recall (<92%) on the external validation dataset. The model could be further improved by efforts to predict the subtype of breast cancer as well as its progression to advanced stages or metastasis. The present model has been deployed as a web-service at https://apalania.shinyapps.io/brcadx/ for non-commercial use. The ideas used in our study could be useful in developing elegant, interpretable AI-assisted diagnostic models for many other cancers and disease conditions, promoting effective decision support aid to medical diagnosis.

## Data Availability

The original contributions presented in the study are included in the article/Supplementary Material, further inquiries can be directed to the corresponding author.
